# The impact of economic rationalization, prioritization and rationing on job satisfaction, motivation and team cohesion in hospitals: a survey among retired physician executives in Germany

**DOI:** 10.1186/s13037-016-0119-4

**Published:** 2017-01-17

**Authors:** Joerg Schnoor, Elmar Braehler, Mohamed Ghanem, Christoph E. Heyde

**Affiliations:** 1 Department of Anesthesia and Intensive Care Medicine, University Hospital Leipzig, Liebigstraße 20, Leipzig, 04103 Germany; 2 Department of Medical Psychology and Medical Sociology, University Hospital Leipzig, Leipzig, Germany; 3 Department of Orthopedics, Traumatology and Plastic Surgery, University Hospital Leipzig, Liebigstraße 20, Leipzig, 04103 Germany

**Keywords:** Economization, Rationalization, Prioritization, Rationing, Job satisfaction, Team cohesion

## Abstract

**Background:**

The growing economization of the health care system and implication of market principles in the medical field have risen new and serious questions on the meaning of the medical profession, the doctor-patient relationship and the orientation of medicine itself. The impact of the dynamic clinical structures on the doctor-doctor and the doctor-patient interaction appear even unpredictable. Therefore, the impact of market-based methods, i.e. rationalization, prioritization and rationing, on job satisfaction, motivation and team cohesion should be quantified.

**Methods:**

The experiences of former and now retired physician executives in numerous hospitals in Saxony were determined. For this purpose, an anonymously written survey using a standardized questionnaire was conducted in the first quarter of 2016.

**Results:**

Rationalization measures were confirmed by 88% of respondents. In more than a third of cases, former executives also experienced prioritization and rationing. The impact of these management techniques on job satisfaction, motivation and team cohesion was carried out in a differentiated manner. There was a tendency to regard rationalization and prioritization measures indifferently to rather disadvantageous, while rationing was predominantly rated negatively.

**Conclusions:**

In addition to rationalization, prioritization and rationing measures have now been part of working strategy at the hospitals. On one hand, the conceptual distinction between the terms still seems imprecise; on the other hand, a creeping and imperceptible medico-ethical transgression of the prioritization to rationing seems to have already taken place.

## Background

Bureaucratization, legalization and economization are three main features of the development of medicine in Germany, comparable to other developed countries [1–3]. The almost daily experienced finite nature of resources is the consequence of scarce social funds, which is exacerbated by the cost of medical progression [4, 5]. At the same time, the economization of medicine threatens quality of results and patient safety [6, 7]. The extent, to which these stresses lead to an impairment of the patient genuinely benefit oriented medicine, is part of the resulting controversy [8–10]. In clinical practice, it has long been a challenge to maintain a balance between ethical and economic goals. This is shown in fundamental debates about medical self-understanding, doctor-patient relationship and the foundations of medicine as such [11–13].

Terms such as prioritization, rationalization and rationing have representative meaning in this debate [14–16]. Although they appear conceptually defined, it remains questionable to what extent these measures are distinguishable from each other in practice. Physicians should actively and creatively contribute to the discourse [11, 13]. For this, the perspective of those physicians and surgeons who were in leading positions in hospitals and thus witnessed the development phase after the implementation of Diagnosis Related Groups (DRG) is crucial. Their experiences with the measures of rationalization, prioritization and rationing are shown in a questionnaire and the impact on the parameters of job satisfaction, motivation and team cohesion are analyzed.

## Methods

A questionnaire-based anonymous study was designed by an interdisciplinary group of medical professionals. We contacted retired executives from hospitals in Saxony (Germany) that had held a senior position in the period from 2010 to 2015 and thus at the time of introduction and adaptation of the DRG system. Correspondence was held through the Medical Syndicate of Saxony (MSS). The Ethics Committee of the University of Leipzig approved this study (404–15–16112015).

### Questionnaire

**Fig. 1:**
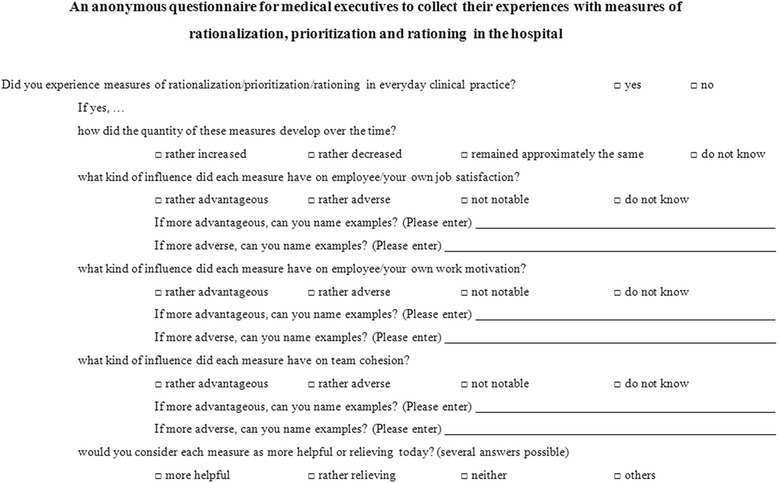
Questions on rationalization, prioritization, rationing, and response possibilities using a 2 to 4-stage Likert scale (response possibilities: more advantageous, rather disadvantageous, no notable, do not know) or partly with a free-fall possibility)

Questions were designed to enquire changes in the period in which measures of rationalization, prioritization and rationing had taken place. Questions had to be simply answered with “yes” or “no”. One particular “yes” response was followed by further questions with answers according to a three— to five-point Likert scale (Fig. 1).

The postal delivery to the home addresses of the addressees (active executives in period between 2010 and 2015) from the fields of internal medicine, gynecology, surgery and anesthesia was performed by the MSS in January 2016. Thus, the collected data was analyzed anonymously.

The return of questionnaires was carried out using pre-fabricated and stamped envelopes directly to the corresponding author. The anonymity of the participants was ensured. In mid-February, a reminder was carried out, again through the MSS. Data was collected until March 31^st^, 2016.

### Terminology

The solicited measures were defined as follows [15, 17, 18]:

1. Rationalization referred to increasing the efficiency of the measures used, thus dispensing with ineffective or less effective measures when compared to alternatives/cost-intensive measures. These measures aim at increasing the level of care while maintaining constant financial expenditure or keeping the level of care while lowering costs. Thus, necessary or useful measures are not withheld from patients.
2. Prioritization is the explicit statement of priority actions or patient groups before others. This creates a multi-level ranking series in which not only methods but also illness and disease groups, supply targets and indications can be triaged. These rankings reflect performance, objectives or evaluation criteria in relation to the provision of medical services.
3. Rationing is the systematic and actual withholding of necessary or useful medical services out of scarcity reasons. In this context, implicit and explicit rationing is distinguished. Rationing can be explicit through a transparent control or implicit by a decision of the doctors at the micro level.

### Outcome parameters

Evaluated data set includes the following questions:

- return rate,
- characteristics of the former executives, departments and hospitals,
- experiences with rationalization, prioritization and rationing,
- impact of the measures on work motivation and satisfaction of their own as well as on the employees,
- impact of the measures on team spirit and cohesion,
- helpfulness for respondents.

## Results

A total of 111 former executives were contacted by the MSS. With 27 answered questionnaires, the response rate was 24%. Two questionnaires were not evaluated because the two respondents did not belong to the defined specialties or hold to the time schedule. Thus, 25 questionnaires were included in the analysis.

The group of executives was composed of 14 former chairpersons and 11 seniors. 23 executives (92%) exercised their managerial function for at least ten years. Two participants exercised their managerial function for a period of six to ten years. The distribution of the different specialties and departments demonstrates Table 1.

**Table 1:** Number of respondents per field of medical activity, number of hospital beds, hospital authorities (pri = private; pup = public; ecl = ecclesiastical), number and direction of hospital volume change

	Number of respondents n (%)	Number of hospital beds (median)	Hospital authorities (pri/pub/ecl)	Hospital volume change (n) (from - > to)
Internal Medicine	10 (40)	100–300	3/4/2	1 pub - > pri
Surgery	7 (28)	100–300	3/4/0	3 pub -> pri (2×); pri - > pri
Gynecology	2 (8)	100–300	0/1/1	0
Anesthesia	5 (20)	300–600	1/1/3	4 pub - > pri (2×); ecl - > pri; pub - > ecl
no response	1 (4)			

### Rationalization measures

Eighty-eight percent (22/25) of respondents reported having experienced rationalization.

- Job satisfaction and motivation

**Fig. 2:**
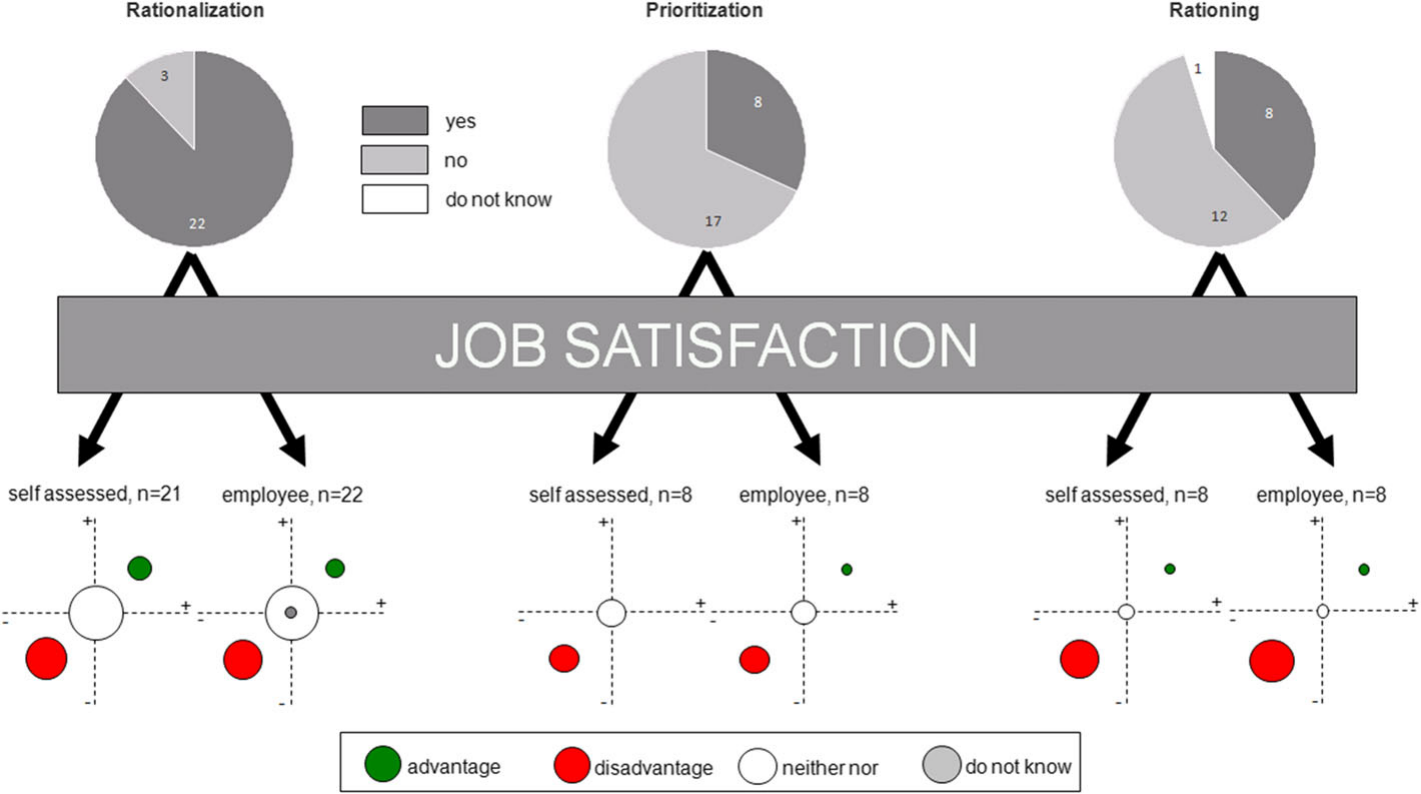
Impact of the measures on the job satisfaction (itself and employees). The upper half gives the number of respondents and the distribution. The lower half shows the effects of the measures on job satisfaction. The corresponding circular sizes indicate the quantitative distribution

Here, rationalization appeared to have brought personal advantage in 14% (3/21) of cases and more advantage concerning job satisfaction of employees in 9% (2/22). No significant impact on job satisfaction was experienced in the self-assessment of 57% (12/21) of respondents and in 64% (14/22) in the assessment of the employees. On the contrary, rationalization measures had rather detrimental impact on job satisfaction in 29% (6/21) cases and in 23% (5/22) concerning employees (Fig. 2).

**Fig. 3:**
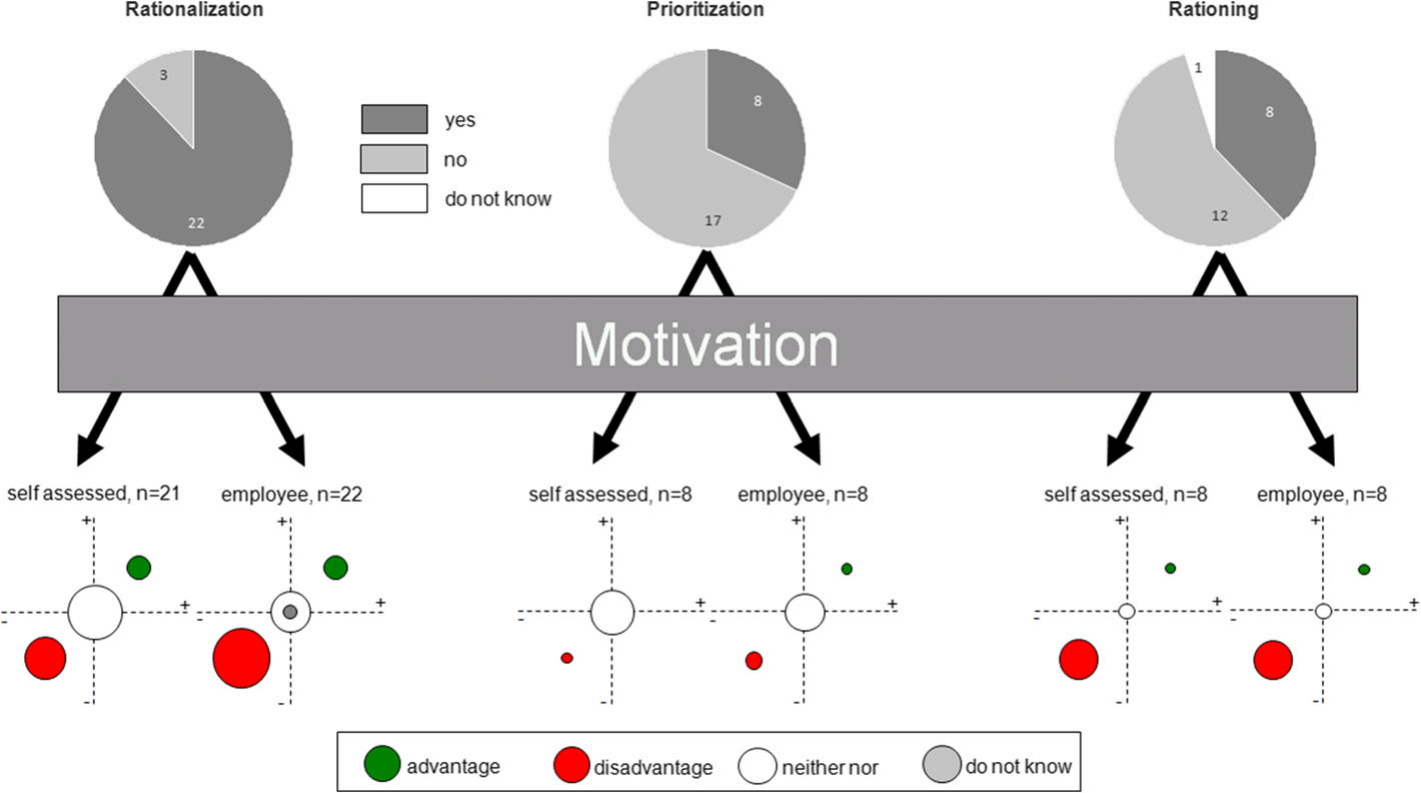
Impact of the measures on work motivation (itself and employees). The upper half gives the number of respondents and the distribution. The lower half shows the effects of the measures on motivation. The corresponding circular sizes indicate the quantitative distribution

The personal work motivation and the employees’ were positively influenced by rationalization measures in 14% (3/21 or 3/22) of the cases. No effect on motivation was experienced on the personal level in 57% (12/21) of cases and in 23% (5/22) of cases concerning employees. On the contrary, rationalization seemed to have lowered personal motivation in 29% (6/21) and motivation of employees in 59% (13/22) (Fig. 3).

- Team spirit and cohesion

**Fig. 4:**
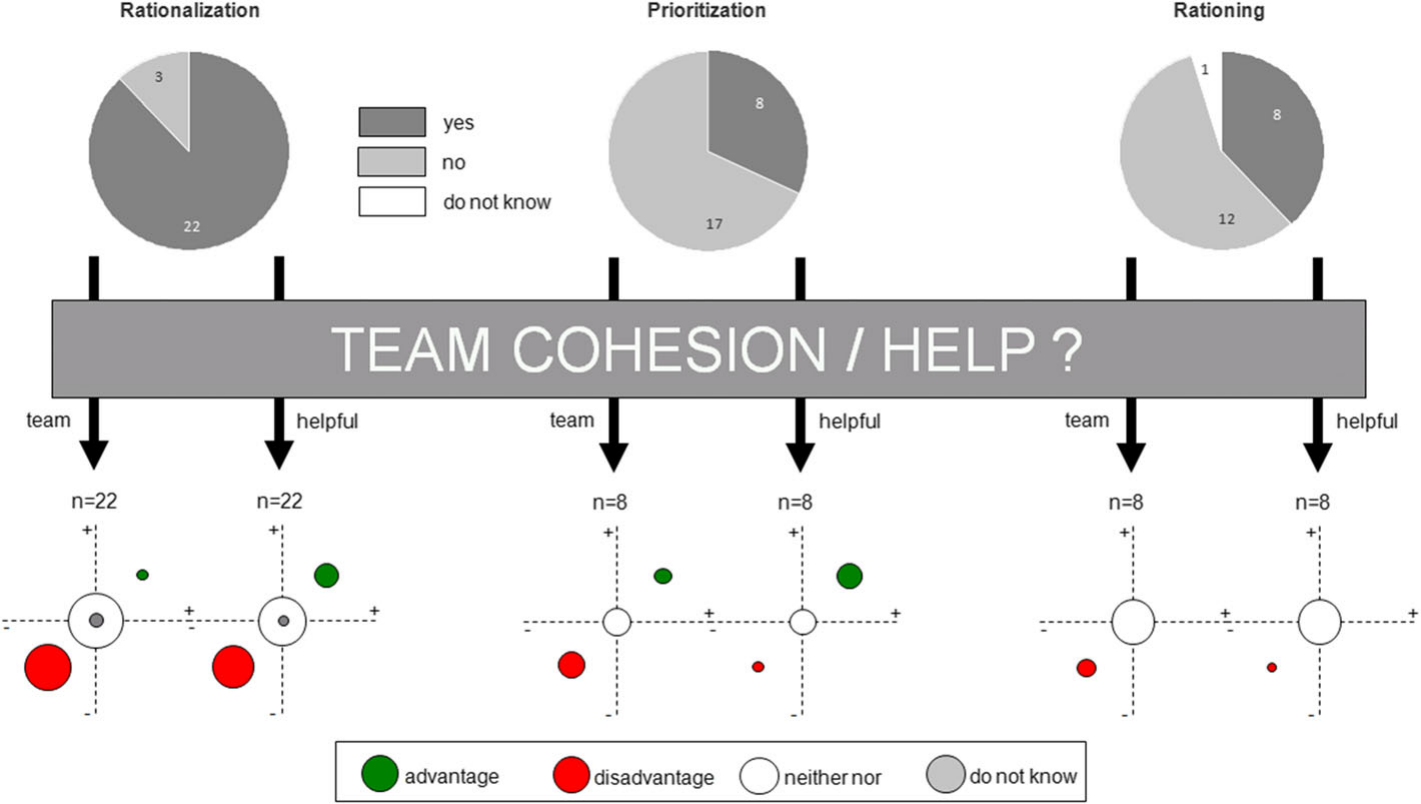
Impact of the measures on team cohesion and the assessing whether the measures have been found helpful. The upper half gives the number of respondents and the distribution. The lower half shows the effects of the measures. The corresponding circular sizes indicate the quantitative distribution

Team cohesion was increased in 5% (1/22) of cases. In 55% (12/22) of cases rationalization could not significantly affect team cohesion. In 36% (8/22) of cases, such measures seemed to have decreased team spirit and cohesion. Rationalization measures were helpful to 14% (3/22) and relieving to 27% (6/22). For 45% (10/22) of participants rationalizations were neither helpful nor relieving (Fig. 4).

### Prioritization measures

Thirty percent (8/25) of respondents reported having experienced prioritization measures.

- Job satisfaction and motivation

None of the colleagues regarded these measures as beneficial on their job satisfaction. In one case (13%) is prioritization seemed have had a positive effect on employees’ satisfaction. No significant effect of this measure was reported in 50% (4/8) with regards to the personal satisfaction and in 38% (3/8) with regards to the employees’ satisfaction. In 50% of each case (4/8) prioritization showed a more adverse effect on personal satisfaction and the employees’ (Fig. 2).

Personal work motivation could not be enhanced by prioritization in any personal case. Concerning employees, work motivation was enhanced in only one case (13%). On the contrary, 88% (7/8) of respondents felt no significant effect of prioritization on work motivation on the personal level and for their employees in 63% (5/8) cases (Fig. 3).

- Team spirit and cohesion

The team cohesion was positively influenced in 25% (2/8) of cases. In 38% (3/8) of cases, it was not significantly affected, while prioritization reported to have had negative impact on team cohesion in equally (38%) of cases. Prioritization measures were helpful or relieving in 43% (3/7) and 14% (1/7) of cases respectively. For 43% (3/7) of respondents prioritization was neither helpful nor relieving (Fig. 4).

### Rationing measures

Thirty-two percnt (8/25) of respondents reported having experienced rationing. 63% (5/8) of them were those respondents who already also experienced prioritization.

- Job satisfaction and motivation

For 13% (1/8), these measures seemed to have had positive impact on their own job satisfaction and the employees’. 25% (2/8) of respondents felt no significant impact of rationing on their personal satisfaction. A more adverse effect was reported in 63% (5/8) of cases. In 75% (6/8) of cases, rationing showed more adverse effects on employees’ satisfaction (Fig. 2).

Rationing measures rather had positive impact on personal work motivation in 13% (1/8) of cases and equally in 13% (1/8) of cases concerning employees (Fig. 2). Respondents in 25% (2/8) of cases experienced no effect. On the contrary, rationing seemed to have had adverse effect on personal work motivation in 63% (5/8) and equally on the employees’ (Fig. 3).

- Team cohesion

No positive impact of rationing on team cohesion was reported in any of the cases. 88% (7/8) of respondents experienced no significant effect on team cohesion. In 13% (2/8) rationing measures had rather a negative effect on team cohesion. Rationing measures seemed to be not regarded as helpful. A relief was experienced by 13% (1/8) of respondents. For 88% (7/8) of respondents rationing proved to be neither helpful nor relieving (Fig. 4).

## Discussion

The data obtained in the course of this survey show that rationing was carried out along with economically oriented measures within the time period of investigation. They also show that original and essential foundations of medicine — work motivation and team cohesion — can be liable to measures of rationalization.

The medical field needs an economic orientation. According to Maio [3], economics is needed where it helps medicine to reach its genuine medical objectives without dissipation. Market-based techniques should only be adapted as long as they do not contradict genuine medical objectives. For this purpose, the economic elements must not only be clearly defined, but they must be without detriment to the patient, to the doctor and to the doctor-patient relationship [2, 9]. For many years, measures of rationalization, prioritization and rationing were discussed in debates. Further, they were not always clearly distinguished from each other in everyday use [19, 20].

### Rationalization

Rationalization is considered an economically useful instrument. The “exhaustion” of this measure and its often-experienced single time effect is regarded as limitation [18]. For the period between 2010 and 2015, the majority of executives surveyed confirmed that rationalization measures were carried out in their clinics. The low percentage without rationalization measures could indicate that these clinics have already experienced prior full rationalization measures. In the majority of cases, rationalization had rather limited effect on job satisfaction. The motivation of the staff seemed to have even been negatively influenced. Possible reasons appear to have been an increased amount of documentation and less available time for patients. In particular, the resulting lack of time in patient contact appears to be a characteristic of the influence of economic measures on medicine [21, 22]. Especially, a decrease of interpersonal affection promises a lucrative cost reduction, since interpersonal affection is irrelevant in a lump-sum payment system. Consequently, the “dictates of the market” was transformed into “dictates of current economics” setting limits to medical and nursing action on patients [3, 19]. Rationalization was also associated with an increased workload and increased absenteeism. This denotes that rationalization was mostly perceived to be less motivating, not promoting team cohesion and generally unhelpful.

### Prioritization

The discussion on prioritization in health care is now regarded as an opportunity to achieve an cost reduction taking the affordability of health care into account [13, 22–24]. Therefore, the most important design criteria were outlined, such as the formulation of an objective medical necessity, the fulfillment of urgent requirements and the maintenance of the solidarity principle [25]. However, social consensus is considered mandatory in a transparent definition and distribution of services [4, 15, 23].

Around one third of respondents reported prioritization measures in their clinics, but only in the departments of Internal Medicine and Anesthesiology. In Surgery and Gynecology, prioritization measures were lacking for some reason that could not be determined regarding the requested data. The impact on job satisfaction appeared to be rather slightly disadvantageous. Motivation, however, was not affected. Team cohesion suffered in around a third of cases. This might be explained by the rather low overall response rate compared to the rationalization measures. On the other hand, enquiry concerned the period of years in which discussion on prioritization has only just begun. Therefore, we can only speculate on the extent of prioritization measures at that time.

### Rationing

Rationing measures are still being perceived as having predominantly negative impact [16, 18]. After all, rationing entails withholding medically necessary and/or useful measures. Yet, about one third of respondents experienced these measures and confirmed a predominantly negative impact on job satisfaction and motivation. Rationing was not perceived as helpful. However, rationing was generally reported to have had no effect on team cohesion. Reifferscheid et al. determined as part of a nationwide survey of senior physicians an even higher rate of 46% [26]. Nationwide, rationing was considered in all specialties of medicine, albeit to a lesser extent, to have been employed lower expenses. Nevertheless, according to Raspe and Schulze rationing is clearly stated to be not accepted in the medical field [16].

Around one third of executives surveyed stated that the measures of rationalization, prioritization and rationing were not conforming to the mission statement of their hospital [20]. In addition, the evaluation of prioritization and rationing showed some overlap. The Central Ethics Commission of the German Medical Association clearly defined the terms and placed them in a comprehensible context, yet it is still not clear whether the two measures are so well distinguished in practice [16, 18, 24].

The main limitation of this study lies in the small number of cases. This mainly appears to be due the small number of retired colleagues investigated within the period between 2010 and 2015 in Saxony. Saxony is one of 16 federal states in Germany with more than four million inhabitants and 78 hospitals [27, 28]. As a result, only 111 former executives were eligible and contacted. Nevertheless, the response rate to the non-incentivized questionnaire study appears high. The selected specialties referred to clinics of basic and standard care, which are basically concerned with the management of a large number of patients in Germany. A nationwide survey would promise results that are more valid. The basic issue, however, remained unsettled.

## Conclusions

Rationalization, prioritization and rationing were carried out along with economically oriented measures. Each prioritization of an issue conditionally automatically posteriorizes the other. Given the finite nature of resources, each posteriorization can easily lead to rationing. A transition of the prioritization into rationing appears to be insidious and is probably not always clearly identifiable in clinical practice. This study provides a solid ground for further discussions, especially to direct future healthcare service to patient welfare and benefit.

## Abbreviations

DRG: Diagnosis Related Groups
MSS: Medical Syndicate of Saxony, Germany
